# Improving deceased donor kidney utilization: predicting risk of nonuse with interpretable models

**DOI:** 10.3389/frai.2025.1638574

**Published:** 2025-08-13

**Authors:** Ruoting Li, Sait Tunç, Osman Y. Özaltın, Matthew J. Ellis

**Affiliations:** ^1^Department of Critical Care Medicine, University of Pittsburgh, Pittsburgh, PA, United States; ^2^Grado Department of Industrial and Systems Engineering, Virginia Tech, Blacksburg, VA, United States; ^3^Edward P. Fitts Department of Industrial and Systems Engineering, North Carolina State University, Raleigh, NC, United States; ^4^Division of Nephrology, Department of Medicine, Duke University School of Medicine, Durham, NC, United States

**Keywords:** deceased donor kidney, kidney transplantation, nonuse risk prediction, predictive modeling, clinical decision-making

## Abstract

**Background:**

Many deceased donor kidneys go unused despite growing demand for transplantation. Early identification of organs at high risk of nonuse can facilitate effective allocation interventions, ensuring these organs are offered to patients who could potentially benefit from them. While several machine learning models have been developed to predict nonuse risk, the complexity of these models compromises their practical implementation.

**Methods:**

We propose simplified, implementable nonuse risk prediction models that combine the Kidney Donor Risk Index (KDRI) with a small set of variables selected through machine learning or transplantation expert input. Our approach also account for Organ Procurement Organization (OPO) level factors affecting kidney disposition.

**Results:**

The proposed models demonstrate competitive performance compared to more complex models that involve a large number of variables while maintaining interpretability and ease of use.

**Conclusion:**

Our models provide accurate, interpretable risk predictions and highlight key drivers of kidney nonuse, including variation across OPOs. These findings can inform the design of effective organ allocation interventions, increasing the likelihood of transplantation for hard-to-place kidneys.

## 1 Introduction

Kidney transplantation is the gold standard treatment for patients with end-stage renal disease. Nearly 80% of kidneys are recovered from deceased donors, however a significant challenge remains: almost 90,000 U.S. patients stay on the waiting list, while *one out of every four* donated kidneys that are recovered for transplantation go unused (Cooper et al., 2019; Lentine et al., 2022; Li et al., 2021). Perceived organ quality plays a crucial role in this alarming nonuse rate, as does the intricacies of appropriately matching available organs with suitable recipients (Mohan et al., 2018). To alleviate such significant discrepancies, mechanisms to expeditiously match donated organs at higher risk of nonuse with patients who may potentially benefit from receiving them emerge as a pressing need (Schold et al., 2019; Stratta, 2022).

In current practice, to increase the utilization and expedite the placement of “hard-to-place” kidneys, organ procurement organizations (OPOs) can deviate from the match-run process and extend out-of-sequence offers to transplant centers. The prevalence of such offers has recently increased, in part due to the latest updates in the kidney allocation system, which inadvertently created delays in local kidney placements (Adler et al., 2021). The lack of transparency and consistency in these discretionary practices has also raised public concerns; a recent New York Times article underscored how such *ad hoc* decisions can erode trust in the system (Rosenthal et al., 2025). Without defined guidelines, allocation exceptions may amplify the existing inequalities in organ access (Hanaway et al., 2020; Lynch and Patzer, 2019). Thus, the main motivation of this study pertains to enabling equitable and transparent allocation decisions by predicting kidneys that require additional effort or interventions for successful placement.

The Kidney Accelerated Placement (KAP) initiative, launched in July 2019, aimed at identifying hard-to-place kidneys and channeling them to transplant centers with a history of accepting such organs (Cooper et al., 2019; Schold and Segev, 2012). However, the KAP project failed to increase organ utilization due to: (i) a vague definition of “hard-to-place” kidneys, and (ii) delayed acceleration of placement for such kidneys until they had been rejected by multiple local and regional transplant programs (Noreen et al., 2022). Our study addresses these two shortcomings by proposing machine learning models that can accurately identify kidneys at high risk of nonuse either *before the beginning* of the match run process or during its *early stages*, enabling *timely interventions*.

**Figure 1:**
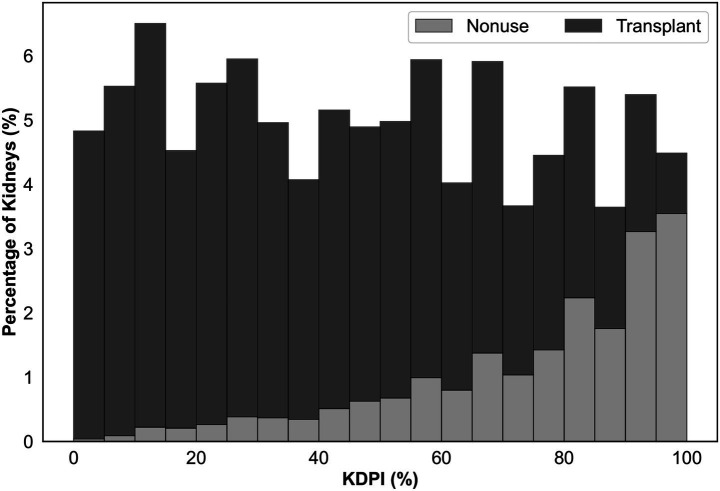
Percentage of deceased donor kidneys recovered for transplantation between 2016 and 2021 that are not utilized (light gray) or transplanted (dark gray) with respect to KDPI.

The Kidney Donor Risk Index (KDRI) and Kidney Donor Profile Index (KDPI) serve as mainstays for clinicians and transplant decision-makers for evaluating kidney quality and predicting post-transplant longevity, both of which subsequently impact the likelihood of offer acceptance (Noreen et al., 2022; Organ Procurement and Transplantation Network, 2020). KDRI quantifies relative graft failure risk, and KDPI maps KDRI onto a cumulative percentile scale. Although originally developed to predict post-transplant outcomes, these indices have also been used by policymakers as proxies for assessing nonuse risk when designing interventions to reduce organ nonuse rates (Dahmen et al., 2019; Organ Procurement and Transplantation Network, 2021). The KAP project, for example, leveraged minimum 80% KDPI (slightly deviating from the conventional 85% threshold) as a primary criterion for triggering accelerated placement interventions (Organ Procurement and Transplantation Network, 2021). However, using KDPI alone for predicting non-utilization is fallible since KDPI was not designed for this purpose. As illustrated in Figure 1, many kidneys with high KDPI find recipients, while a significant portion with KDPI < 85% go unused.

We propose implementable and intuitive machine learning models to predict the risk of kidney nonuse during the match run. Rather than disregarding KDRI, a widely adopted and clinically trusted metric, our simplified risk models leverage its established acceptance, repurposing it for accurate nonuse prediction by combining it with a limited number of additional variables. Our findings demonstrate that this integration significantly improves predictive accuracy and interpretability, aligning closely with current transplant practices. Additionally, we develop comprehensive risk models as benchmarks, deliberately excluding KDRI to avoid potential inherent biases or limitations associated with this metric, and incorporating all relevant variables without constraints. Our computational experiments highlight the competitive performance of our proposed simplified models, identifying key donor- and OPO-level factors that influence the utilization of hard-to-place organs. These proposed models hold promise for improving kidney utilization rates by enabling timely, targeted placement interventions.

The remainder of this paper is organized as follows: Section 2 reviews the related work. Section 3 describes the dataset and modeling methodology. Section 4 presents the experimental results. Section 5 discusses key findings, limitations, and ethical considerations, and Section 6 concludes the paper.

## 2 Related work

Previous research on kidney nonuse has primarily relied on predictive modeling methods, leveraging donor and organ characteristics to anticipate the likelihood of organ nonuse. Early foundational studies introduced logistic regression models to estimate nonuse probabilities. Massie et al. (2010) developed the Probability of Nonuse or Delay (PODD), which predicted whether kidneys would either remain unused or experience extended cold ischemia time. Building upon this, Marrero et al. (2017) presented a logistic regression model that achieved improved predictive performance compared to the KDPI, demonstrating the potential of predictive analytics to improve organ allocation decisions. Zhou et al. (2019) further showed that directing high-PODD kidneys to transplant centers more inclined to accept them could significantly improve their utilization. A complementary study Cohen et al. (2018) identified non-quality-related factors, such as procurement timing (weekends, holidays) and local waiting list characteristics, as critical variables influencing kidney acceptance.

More recently, researchers have increasingly employed advanced machine learning (ML) and natural language processing methods to improve nonuse predictions. Sageshima et al. (2024) effectively combined structured donor data with unstructured donor narratives to identify kidneys at higher non-utilization risk. Similarly, Barah and Mehrotra (2021) developed random forest and gradient boosting models leveraging detailed donor information available at the match-run onset, achieving strong predictive outcomes. However, despite their accuracy, these advanced ML models are often regarded as “black-box” methods; limited in interpretability due to their complexity and reliance on numerous input variables (Linardatos et al., 2021; Rudin, 2019). Moreover, they did not include KDRI, a metric widely employed by clinicians and policymakers to assess organ quality.

A critical limitation across much of the existing literature is the exclusive focus on donor and organ-level predictors, while neglecting broader system-level influences, notably the critical role of OPOs. Recent evidence highlights significant variability among OPOs in procurement practices and performance, with direct implications for organ utilization (Concepcion et al., 2023; Doby et al., 2022). Ignoring OPO-specific characteristics may lead to models missing important determinants of kidney nonuse and, consequently, diminish their applicability.

Our study contributes to existing literature in the following distinct ways. First, addressing the interpretability gap of previous ML-based approaches, we develop simplified predictive models utilizing a minimal and carefully selected subset of clinically relevant variables. This approach promotes transparency and clinical practicality, facilitating direct application by decision-makers. Second, rather than disregarding established metrics like KDRI, we leverage its clinical acceptance, integrating and repurposing it within our predictive framework. This strategy improves both the accuracy and usability of nonuse risk assessment while aligning closely with existing clinical practices. Finally, we explicitly integrate OPO-level characteristics by clustering OPOs based on their kidney utilization performance. By doing so, we move beyond purely donor-centric views, acknowledging and modeling critical system-level factors that influence kidney utilization outcomes.

## 3 Materials and methods

### 3.1 Data

Our data set, provided by the United Network for Organ Sharing, includes records from 61,320 deceased donors, between January 2016 and September 2021, who had at least one kidney recovered for transplantation. Each record contains 530 variables, encompassing donor demographic characteristics, physical properties, and relevant medical information, such as laboratory values and comorbidities. Furthermore, we obtained Potential Transplant Recipient (PTR) data for the same time period, which captures all kidney offers made to patients on the US waiting list. The PTR data logs the match run creation time for each donor, which was used to determine ischemia time. We removed 110 donors missing a match run creation time from the analysis.

We identified an initial list of variables linked to kidney nonuse in the literature (Barah and Mehrotra, 2021; Marrero et al., 2017; Massie et al., 2010). This list was further augmented by kidney transplant experts on our team, leading to 36 variables included in our analysis (Table 1). These variables were used to generate an observation vector for each kidney, where each kidney from the same donor was treated as a separate observation (Supplementary Figure A1).

**Table 1:** The results of the univariate analysis (*N* = 117,747 kidneys).

Variable name	Category	Mean/percent	Odds ratio	95% CI
Age (year)		40.48	1.07	1.07, 1.07
Height (cm)		169.33	1.00	0.99, 1.00
BMI (kg/m^2^)		28.37	1.03	1.03, 1.04
Creatinine (mg/dL)		1.43	1.39	1.38, 1.40
Warm Ischemia Time (h)		0.01	2.30	2.00, 2.65
Initial Cold Ischemia Time (h)		0.23	1.06	1.05, 1.07
KDRI		1.35	16.50	15.90, 17.10
Ethnicity	Other	68.83%		
	Asian	2.52%	1.14	1.04, 1.25
	African American	14.40%	1.23	1.18, 1.28
	Hispanic	14.25%	0.80	0.76, 0.83
Blood type	O	47.75%		
	AB	3.42%	1.32	1.23, 1.42
	B	11.65%	1.05	1.00, 1.10
	A	37.18%	1.04	1.01, 1.08
Cause of death	Other	3.17%		
	CVA/Stroke	25.44%	1.86	1.71, 2.02
	Anoxia	43.82%	0.89	0.82, 0.97
	Trauma	27.57%	0.47	0.43, 0.51
OPO clusters	Cluster 1	43.76%		
	Cluster 2	24.67%	0.83	0.80, 0.86
	Cluster 3	6.14%	0.72	0.67, 0.77
	Cluster 4	24.33%	1.34	1.30, 1.39
	Cluster 5	1.10%	0.83	0.72, 0.96
Diabetes status	Yes [>0 Years]	10.66%	4.63	4.45, 4.81
	Yes [>5 Years]	5.24%	5.78	5.49, 6.09
	Yes [>10 Years]	3.19%	6.47	6.05, 6.92
Glomerulosclerosis	>5	20.44%	8.13	7.87, 8.39
	>10	11.96%	12.10	11.60, 12.50
	>15	7.89%	18.00	17.10, 18.90
	>20	5.41%	26.30	24.60, 28.20
Interstitial fibrosis level	Absent	68.65%		
	Minimal	14.77%	2.84	2.73, 2.95
	Advanced	16.58%	7.53	7.26, 7.80
Vascular changes level	Absent	74.15%		
	Minimal	10.57%	2.66	2.55, 2.78
	Advanced	15.28%	6.18	5.97, 6.40
Biopsy indicator	No	44.93%		
	Yes	55.07%	6.48	6.24, 6.74
Gender	Male	61.12%		
	Female	38.88%	1.35	1.31, 1.39
Dual kidney	No	97.95%		
	Yes	1.04%	2.72	2.42, 3.05
Enbloc kidney	No	98.99%		
	Yes	1.01%	0.77	0.65, 0.89
DCD donor	No	76.47%		
	Yes	23.53%	1.21	1.17, 1.25
History of cancer	No	96.97%		
	Yes	3.03%	2.68	2.50, 2.87
History of smoking	No	78.67%		
	Yes	21.33%	2.11	2.04, 2.18
History of hypertension	No	67.06%		
	Yes	32.94%	4.35	4.22, 4.48
History of myocardial infraction	No	95.86%		
	Yes	4.14%	3.16	2.98, 3.35
History of cocaine	No	77.43%		
	Yes	22.57%	0.86	0.83, 0.89
History of I.V. drug	No	86.95%		
	Yes	13.05%	0.77	0.73, 0.80
History of other drugs	No	51.93%		
	Yes	48.07%	0.63	0.61, 0.65
Insulin	No	64.34%		
	Yes	35.66%	1.23	1.20, 1.27
CMV status	Negative	38.37%		
	Positive	61.63%	1.23	1.19, 1.26
HBV core antibody status	Negative	95.17%		
	Positive	4.83%	2.39	2.26, 2.53
Risk for blood-borne disease transmission	No	75.14%		
	Yes	24.86%	0.76	0.73, 0.79
Protein in urine	No	48.45%		
	Yes	51.55%	1.45	1.41, 1.49
HCV NAT results	Negative	94.57%		
	Positive	5.43%	1.75	1.65, 1.84
Arginine vasopressin with 24 h pre-clamp	No	41.06%		
	Yes	58.94%	0.59	0.57, 0.61
Coronary angiogram	No	78.79%		
	Yes	21.21%	0.52	0.52, 0.57
Pump	No	60.88%		
	Yes	39.12%	0.86	0.83, 0.88

For continuous variables, the mean value is reported. For binary and categorical variables, the percentage of kidneys in each category is reported. The highlighted categories are used as the reference group when calculating the odds ratio for non-utilization.

### 3.2 Variable creation

We created additional variables using some of the 36 variables. Specifically, we computed Cold Ischemia Time (CIT) and Warm Ischemia Time (WIT) for each kidney, since prolonged CIT and WIT adversely affect graft function (Debout et al., 2015; Kamińska et al., 2016), and transplant centers carefully evaluate these metrics when responding to kidney offers. For kidneys from Donation after Cardiac Death (DCD) donors, we calculated the WIT as the difference between agonal time and the clamp time. We calculated the CIT at the first match run creation (referred to as *CIT onset*) as the gap between the clamp time and the first match run creation time. CIT onset was set to zero if the clamp time is after the first match run creation.

En-bloc kidney transplantation is a procedure where two small kidneys from a donor weighing less than 18 kg are transplanted into one recipient (Organ Procurement and Transplantation Network, 2023). Dual (or 2-for-1) kidney transplantation involves transplanting both kidneys from a donor weighing at least 18 kg, which are individually less suitable for transplantation (Organ Procurement and Transplantation Network, 2023). To account for the disparities between the nonuse risk of en-bloc and dual kidneys, we created two indicator variables, namely *isEnbloc* and *isDual*.

We applied the k-means clustering algorithm (Hartigan and Wong, 1979; Jadlowiec et al., 2023) to categorize OPOs based on two distinct features: (i) the overall percentage of kidneys recovered by each OPO that were successfully transplanted, and (ii) the transplantation percentage specifically among kidneys with KDPI ≥ 85%. To select an appropriate number of clusters, we constructed an elbow plot illustrating average within-cluster Euclidean distances across a range of potential cluster numbers (k values from 2 to 40). Although the elbow plot (Supplementary Figure A2) suggested *k* = 10 as a potential optimal solution, we selected *k* = 5 to achieve a suitable balance between meaningful cluster differentiation and statistical robustness, considering the relatively small number of OPOs. The resulting OPO clusters were then incorporated into our predictive models as categorical indicator variables, enabling us to systematically evaluate the influence of OPO-level behaviors on kidney utilization outcomes.

### 3.3 Missing data imputation and data exclusion

Our data included variables with unspecified categories or categories that were deemed inconsequential to kidney disposition (e.g., the distinction between blood types A, A1, and A2). We consolidated those categories (see Supplementary Table A1). We further processed the data by replacing creatinine values above 20 with 20 for six kidneys; by computing missing BMI values using available donor weight and height; and by imputing missing WIT and CIT onset. After these steps, 1,406 kidneys (1.2%) with missing data were excluded from the analysis.

Both CIT onset and WIT were calculated and imputed at the donor level. Among 14,516 DCD donors, 358 (about 2.5%) had missing WIT, which were imputed using the median of the observed WIT values (0.383). For non-DCD donors, WIT was set to 0. Out of 61,210 donors, 19 had missing CIT onset (~0.03%), which were imputed using the median of the observed CIT onset values (0). Although we did not perform multiple imputation or formal sensitivity analyses due to low missingness of WIT and CIT, more robust imputation techniques may reduce bias and improve model generalizability in datasets with higher missingness.

We excluded two kidneys with more than 24 h WIT as outliers to avoid bias in our results. Furthermore, kidneys that were not used for reasons unrelated to their characteristics were excluded. In particular, we excluded kidneys with nonuse reasons “not as described” (0.05% of kidneys, 60 kidneys), or “recipient determined to be unsuitable in the operating room” (0.06% of kidneys, 76 kidneys). Lastly, categories with less than 20 observations were removed, as detailed in Supplementary Table A1.

### 3.4 Model development for kidney nonuse risk prediction

We initially considered a broad set of candidate models, including logistic regression (with and without splines), decision trees, naïve Bayes, support vector machines (SVMs), random forests, and generalized additive models (GAMs). Our goal was to identify models that would jointly satisfy: (i) strong predictive performance, (ii) interpretability for clinical implementation, (iii) support for flexible variable selection and co-design integration, and (iv) established usage and acceptance in health services research and clinical practice. After evaluating the performance of the initial models, we chose logistic regression (a parametric model) and random forest (a non-parametric model) as our final modeling approaches. Logistic regression was chosen for its simplicity, widespread usage in medical literature and clinical interpretability offering coefficient-based insights into variable effects. To account for non-linear relationships in logistic regression, we used linear splines for continuous variables (see Supplementary Table A2). The random forest model leverages an ensemble of decision trees to capture complex interactions and improve accuracy. It was selected for its superior out-of-sample predictive performance, resilience to overfitting, and robustness to noisy features and non-linear interactions. Using these two models, we developed two classes of nonuse risk prediction frameworks:

**Simplified Risk Models:** These models use KDRI alongside a minimal set of variables, streamlining the risk assessment. For *random forest (RF)* models, initial training was done using all of the original variables (i.e., variables included in the UNOS data and created variables explained in Section 2.2) and KDRI. We then identified the top five, seven, and nine variables based on permutation importance. Models were then re-trained with these variables and KDRI. For *logistic regression (LR)* models, we included both the original and spline variables and compared the coefficients of normalized variables to identify the top ones.

**Comprehensive Risk Models:** Serving as a benchmark, these models excluded KDRI to avoid any implied, intrinsic biases or shortcomings of this metric. The *RF model* used all of the original variables, while the *LR model* used both the original and spline variables. The efficacy of simplified and comprehensive risk models was compared. Additionally, we evaluated the performance of our models against a benchmark nonuse risk prediction model that uses only KDRI, directly correlating it with nonuse risk, which we refer to as *KDRI alone*.

We performed five-fold stratified cross-validation and evaluated performance using the receiver operating characteristic (ROC) and the precision-recall (PR) curves. The area under the ROC curve (AUROC) measures the model’s ability to distinguish between transplanted and unused kidneys. Precision and recall are critical metrics for prediction performance. Precision is equivalent to Positive Predictive Value (PPV); representing the ratio of correctly predicted unused kidneys to the total number of kidneys predicted as unused. Recall is equivalent to sensitivity; representing the ratio of correctly predicted unused kidneys to the actual number of unused kidneys. As there is a trade-off between precision and recall, the proposed models can achieve higher recall, at the expense of more false positives (i.e., transplanted kidneys predicted as unused) by lowering the prediction threshold. We plotted the PR curve for each model to visualize this trade-off and calculated the area under the PR curve (AUPRC).

### 3.5 Prediction scenarios

We developed models to predict kidney nonuse early in the allocation process. While the majority of variables considered in our analysis are available pre-match-run, biopsy-related variables like glomerulosclerosis, interstitial fibrosis, and vascular change usually become available later in the match run. The role of these variables in the kidney offer response decisions can be paramount (Mohan et al., 2018), and more than half of the recovered kidneys undergo biopsy during the match run (Lentine et al., 2019). To support real-world use, we created models both with and without biopsy-related variables. In practice, decision-makers can pivot to the model with biopsy-related variables once biopsy data become available.

### 3.6 Co-design of nonuse risk prediction models

Machine learning models often prioritize variables based on statistical patterns that may not reflect clinical relevance, especially when training data is not fully representative of the broader population (Park et al., 2023). To address this, we conducted a co-design process with kidney transplant experts to enhance model interpretability and clinical utility. The co-design process involved a methodical evaluation and potential substitution of variables within our models. Clinicians reviewed the set of variables determined by the machine learning algorithms. They recommended removing variables they deemed less relevant in kidney utilization. They also recommended a set of variables to be considered in our models for assessing the kidney nonuse risk. We evaluated these variables for their statistical contributions to the prediction performance. The co-design process harmonized the variables selected by machine learning models with the variables deemed important by the clinical experts. This approach aimed to foster greater acceptance and usability of the proposed models.

## 4 Results

### 4.1 Study population and variable analysis

**Figure 2:**
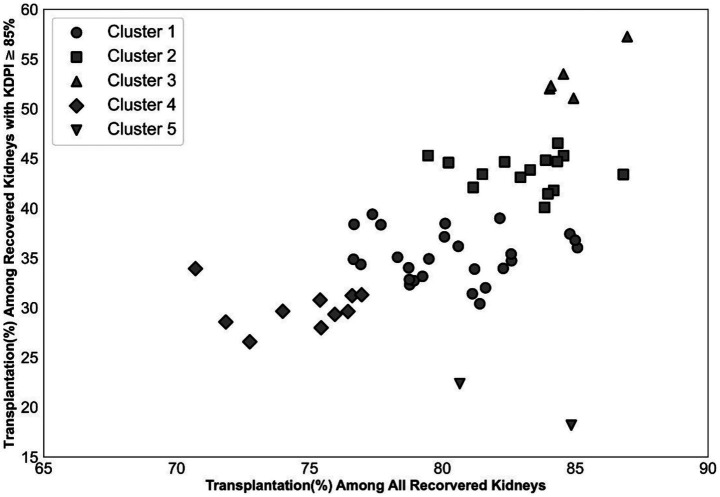
Impact of OPO centers on the disposition of hard-to-place kidneys. Categorizing OPO centers into five clusters to account for their impact.

Supplementary Figure A1 outlines the data preparation process. Starting with 119,334 procured kidneys from 61,210 donors, we implemented four exclusion criteria removing 1,587 kidneys (1.33%). The final dataset consisted of 94,057 transplanted kidneys (79.9%) and 23,690 unused kidneys (20.1%). Figure 2 demonstrates the k-means clustering of the 58 OPOs into five groups based on the transplantation percentage of all kidneys and the transplantation percentage of hard-to-place (KDPI ≥ 85%) kidneys they recovered.

Univariate analysis results (i.e., single factor analysis for nonuse) are displayed in Table 1. All variables exhibited statistically significant odds ratios for non-utilization. For instance, all else equal, with each hourly increment in WIT and CIT onset, we expect to see a 130 and 6% increase in the odds of nonuse as indicated by their odds ratios of 2.30 and 1.06, respectively.

### 4.2 Model prediction performance

**Figure 3:**
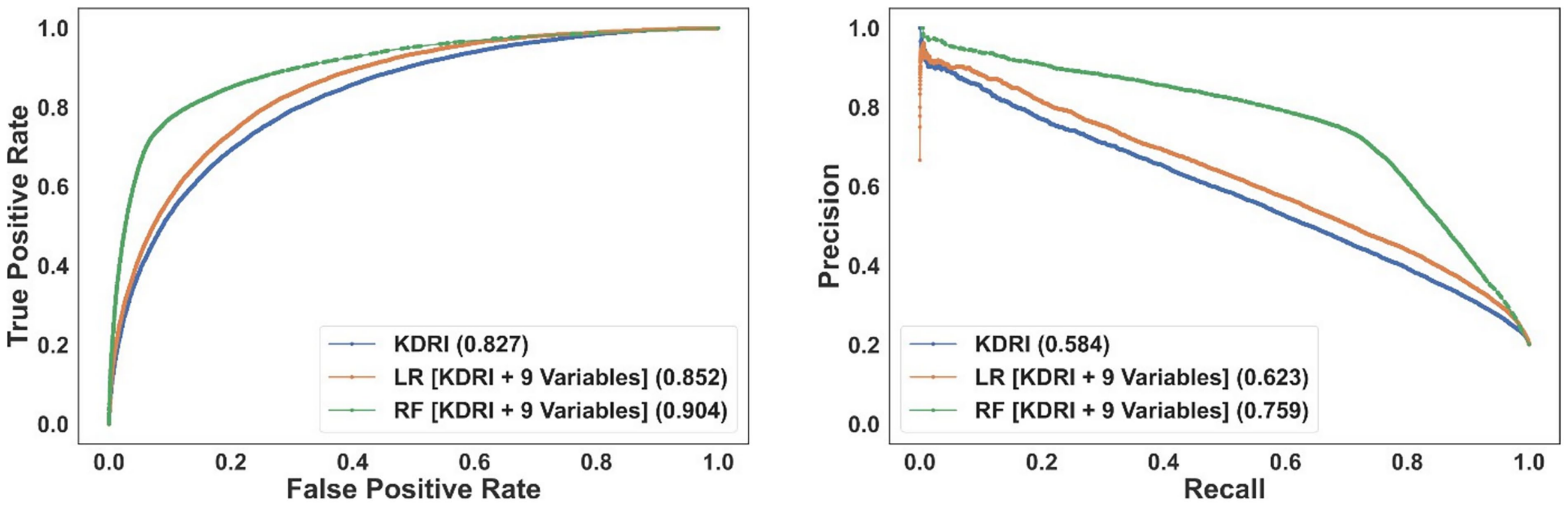
The ROC (left) and PR (right) curves for the simplified models incorporating KDRI and nine additional (non-biopsy-related) variables. The area under the curve of each model is reported in the legend.

Supplementary Figure A3 presents the ROC and PR curves. The models that use KDRI and nine additional variables (*simplified risk models*) matched the performance of models trained on the entire variable set (*comprehensive risk models*). Figure 3 shows the performance of the models in the same plot for comparison. The RF model has the best performance, followed by the LR model-both exhibiting clearly superior performance compared to using KDRI alone. Supplementary Figures A4, A5 show the impact of biopsy results on model performance: the performance of the LR model markedly improves whereas the performance of the RF model remains comparable. Supplementary Table A3 reports the number of false positives avoided by the proposed models compared to using KDRI alone. For example, the RF model can precisely predict and potentially increase the transplantation likelihood of nearly 12,000 unused kidneys at a recall (sensitivity) level of 0.5. Concurrently, this model would prevent the misclassification of over 5,500 transplanted kidneys compared to using KDRI alone, mitigating thousands of needless interventions. The efficacy of the proposed prediction models becomes increasingly evident at higher recall levels.

### 4.3 Selected variables

In the rest of this manuscript, we focus on the random forest model that includes KDRI and nine additional variables since it outperforms the other models, and denote the model without the biopsy information as the baseline model. Table 2 lists the model variables in for both prediction scenarios, with and without biopsy data, ordered by permutation importance. Creatinine, age, BMI, history of smoking, and height are chosen in both scenarios. In the model with biopsy information, glomerulosclerosis, interstitial fibrosis, and the biopsy indicator replaced the history of hypertension, coronary angiogram, and DCD indicator that were selected among the top 10 variables in the model without biopsy information.

**Table 2:** Variable selected by proposed simplified risk models when excluding and including biopsy-related variables.

Baseline model	Model with biopsy information
**KDRI**	**KDRI**
**Creatinine**	**Creatinine**
**Age**	**Age**
History of Hypertension	Glomerulosclerosis
**Arginine**	Biopsy Performed
**BMI**	**BMI**
**History of Smoking**	Interstitial Fibrosis
DCD	**History of Smoking**
Coronary Angiogram	**Height**
**Height**	**Arginine**

Variables that are commonly selected in both scenarios are given in bold.

### 4.4 The role of OPOs in kidney disposition

The clustering analysis in Figure 3 highlights differences in kidney disposition performance among OPOs. Correspondingly, OPO cluster indicators show significant unadjusted odds ratios for nonuse in Table 1. This result might be due to the difference in the percentage of hard-to-place kidneys procured by OPOs in each cluster. To further assess the impact of OPO cluster variables, we computed their odds ratios for nonuse by adjusting for the nonuse risk predicted by the model without biopsy-related variables. Supplementary Table A4 presents these adjusted odds ratios using cluster 1 as the reference. Except for cluster 5, which has fewer observations than other clusters, all other clusters yielded significant risk-adjusted odds ratios for nonuse. For example, kidneys from OPOs in cluster 3 are significantly less likely, while those in cluster 4 are more likely, to go unused compared to an equivalent kidney from cluster 1 with similar projected nonuse risk.

### 4.5 Factors increasing transplantation likelihood of high nonuse risk kidneys

In this section, we analyze factors associated with increased transplantation likelihood among hard-to-place kidneys to inform potential interventions. A kidney was defined as hard-to-place if its predicted nonuse risk exceeded 0.75; a threshold that identified a similar number of unused kidneys as the KDPI 85% benchmark. Specifically, of the 12,916 kidneys identified as hard-to-place, 10,845 (84%) were not used, while 2,071 (16%) were transplanted. We perform a univariate analysis among hard-to-place kidneys by adjusting for the nonuse risk. The results of this analysis are presented in Table 3, spotlighting factors that are associated with a higher transplantation likelihood of hard-to-place kidneys.

**Table 3:** Top significant factors that are associated with increased transplantation likelihood for hard-to-place kidneys.

Variable name	Adjusted odds		Percentage of transplanted hard-to-place	Percentage of transplanted hard-to-place
	Ratio for transplant	95% CI	Kidneys when the variable value is YES	Kidneys when the variable value is NO/Baseline
Enbloc kidney	5.88	1.76, 22.67	63%	16%
Dual kidney	5.31	3.98, 7.07	52%	16%
OPO cluster3 (Baseline: OPO cluster 1)	2.04	1.65, 2.51	28%	16%
Pump	2.01	1.82, 2.21	23%	12%
OPO cluster2 (Baseline: OPO cluster 1)	1.13	1.00, 1.28	19%	15%

The odds ratio is adjusted for the nonuse risk.

### 4.6 Results of the co-design experiment

Kidney transplant experts on our team identified three baseline model variables, history of smoking, coronary angiogram, and height, as less clinically relevant, and proposed six potential alternatives to replace them: OPO cluster, history of diabetes, cause of death, insulin use, protein in urine, and pump use. Similarly, for the model with biopsy information, eight potential variables were suggested (OPO clusters, history of diabetes, cause of death, insulin, protein in urine, pump, history of hypertension, and DCD indicator) to replace the two that were deemed less relevant (history of smoking and height).

We evaluated all combinations of these substitutions (20 alternatives, six choose three) for the baseline model and 28 alternatives (8 choose 2) for the model with biopsy data, and selected the configurations with the highest AUROC and AUPRC. Table 4 lists the final variables selected in these models. The expert-guided modifications did not compromise the predictive performance of our models; the AUC values remained comparable, and even increased slightly, for both the baseline model (0.905 versus 0.904 for AUROC and 0.767 versus 0.765 for AUPRC) and the model with biopsy information (0.905 versus 0.906 for AUROC and 0.771 versus 0.771 for AUPRC).

**Table 4:** Variables of the random forest nonuse risk prediction model after the co-design.

Baseline model	Model with biopsy information
KDRI	KDRI
Creatinine	Creatinine
Age	Age
History of Hypertension	Glomerulosclerosis
Arginine	Biopsy Performed
BMI	BMI
DCD	Interstitial Fibrosis
**OPO Cluster**	Arginine
**Cause of Death**	**OPO Cluster**
**Pump**	**Pump**

Variables that are added to the model during the co-design process are in bold.

Table 5 reports stratified 5-fold cross-validation results for both models given in Table 4. This evaluation approach allows us to assess prediction performance using unseen data partitions. Our dataset from the OPTN captures every deceased kidney donor and every organ offer in the United States and therefore spans all donor service areas, OPOs, allocation practices, and seasons. That breadth reduces spectrum bias and makes the development sample far more diverse than the typical “single-center development/separate-center validation” set-up. Furthermore, we designed our models to be parsimonious and composed of clinically interpretable variables, improving their adaptability across different settings.

**Table 5:** In-sample cross-validation and external validation results of the baseline RF model and the RF model with biopsy information with variables presented in Table 4.

Performance metrics	In-sample cross-validation		External validation	
	Baseline model	Model with biopsy information	Baseline model	Model with biopsy information
Accuracy	0.8929	0.8860	0.8374 (0.0062)	0.8597 (0.0054)
Precision	0.7580	0.7537	0.6614 (0.0214)	0.7223 (0.0229)
Recall	0.6870	0.6439	0.4258 (0.0519)	0.4696 (0.0400)
F1 Score	0.7207	0.6945	0.5157 (0.0366)	0.5680 (0.0308)
AUROC	0.9036	0.9059	0.8349 (0.0075)	0.8565 (0.0096)
AUPRC	0.7653	0.7714	0.6082 (0.0277)	0.6703 (0.0280)

To assess the generalizability of the models, Table 5 presents the results of external validation experiments using a holdout test set. Specifically, we randomly select 10 out of 58 OPOs to serve as the external test group and exclude all kidneys from these OPOs during model training. We then train RF models using the variables listed in Table 4 on kidneys from the remaining 48 OPOs and evaluate model performance on the holdout kidneys from the 10 excluded OPOs. This process is repeated 10 times, each with a different random selection of test OPOs. Table 5 reports the average and standard deviation (in parentheses) of the performance metrics across these 10 iterations.

The baseline model without biopsy information shows slightly better in-sample cross-validation performance. The performances of both models drop in out-of-sample validation tests. However, the model incorporating biopsy information demonstrates superior generalizability, achieving higher out-of-sample validation performance. This suggests that including biopsy data may enhance the model’s ability to perform reliably in real-world clinical settings, where generalizability to new cases is critical.

## 5 Discussion

Our study proposes kidney nonuse risk prediction models consisting of KDRI and nine additional variables. By achieving a balance between simplicity and performance, these models address a crucial gap in the organ allocation system; the need for easy-to-use yet accurate kidney nonuse prediction models. The proposed models can provide transparent and interpretable decision support to initiate interventions and manage allocation exceptions within the match-run system, increasing the transplantation likelihood of hard-to-place kidneys. Our simplified models significantly outperform using KDRI alone in predicting kidney nonuse risk, and exhibit performances on a par with substantially larger models with more variables.

While biopsy results are often considered critical for assessing kidney quality, our findings indicate that incorporating biopsy information does not substantially enhance the performance of the RF models. This suggests that RF models are robust to the absence of biopsy data and can offer reliable predictions even when such information is unavailable; an important feature in time-sensitive allocation decisions. This challenges conventional assumptions in the literature that biopsy data are indispensable for predicting nonuse (Husain et al., 2022; Mohan et al., 2018). In contrast, biopsy variables such as glomerulosclerosis and interstitial fibrosis significantly improve the performance of the LR models, reinforcing the idea that the utility of biopsy data may be model-specific.

In addition to KDRI, terminal creatinine level, age, BMI, and use of arginine vasopressin within 24 h pre-cross clamp are significant predictors of kidney nonuse risk. If biopsy is performed, our models also utilize variables such as interstitial fibrosis and glomerulosclerosis, aligning well with previous studies that emphasized the role of glomerulosclerosis in kidney non-utilization (Kasiske et al., 2014; Reese et al., 2021). If the biopsy results are not available, then the models utilize variables such as the DCD indicator and history of hypertension. It is worth noting that some of the variables used in our models, such as age, creatinine, and history of hypertension are also considered in KDRI calculation. The predictive performance gap between the proposed models and using KDRI alone emphasizes the importance of recalibrating KDRI for nonuse risk prediction.

Our prediction results reveal the significance of OPO-related factors in the utilization of hard-to-place kidneys. By clustering OPOs based on their performance in placing both all kidneys and high-KDPI kidneys, we identified a subset of OPOs that consistently outperformed others in placing hard-to-place organs. The adjusted odds ratios in our analysis confirm that, even after controlling for kidney-level predicted nonuse risk, OPO cluster membership remains a significant predictor of utilization. The inclusion of OPO-related factors in our risk prediction models is not just a technical innovation but a call to action for the transplantation community to analyze and disseminate the successful strategies of high-performing OPOs, thereby elevating overall practice standards and to encourage other OPOs to adopt similar, effective approaches in organ recovery and allocation. For example, the literature documents major disparities in making out-of-sequence kidney offers to accelerate the placement of hard-to-place kidneys (King et al., 2022). Our models can help mitigate such disparities by providing guidance to OPOs for identifying hard-to-place kidneys that can be intervened for better utilization and for standardizing interventions to enhance transparency and equitability.

The results of the model co-design approach confirm that data-driven machine learning methods and clinical expertise are not mutually exclusive but complementary. By incorporating the insights of transplant experts into the model development process, we have created models that not only have high prediction performance but also align well with real-world clinical judgments, enhancing the medical relevance of our results. In particular, three variables that are deemed less relevant to kidney utilization, the history of smoking, coronary angiogram, and height (when considered in addition to BMI), are replaced with OPO cluster, cause of death, and pump indicator variables through the co-design process.

After identifying hard-to-place kidneys in our data using the proposed prediction models, we explored characteristics associated with an increased transplantation likelihood under the current allocation system. These insights can inform the development of both *operational* and *system-level* interventions. Operational interventions that are identified in our analysis include pumping kidneys, which can help maintain graft function (Bathini et al., 2013), and presenting dual offers, which can increase the chances of acceptance (Tanriover et al., 2014). System-level interventions require strategic changes at the policy or organizational level and often involve a longer-term approach. Such interventions identified in our analysis include identifying and promoting the best organ recovery and allocation practices across OPOs or the integration of an effective nonuse risk prediction framework into the national allocation system.

Our study is not without limitations. Our dataset, spanning 2016–2021, may not fully capture the impact of recent policy changes post-March 2021. Recent trends, such as the increasing acceptance of hepatitis C-positive kidneys due to treatment advances (Buchanan-Peart et al., 2023), highlight the need for periodic model retraining or recalibration. While our OPO clustering approach captures meaningful variation across OPO groups, clustering based on organ utilization performance may conflate regional variations in organ availability, listing practices, or patient demographics with OPO-level behaviors, potentially overstating their influence. Moreover, transportation infrastructure differences (e.g., rural vs. urban OPOs, proximity to transplant centers, availability of commercial flights) and differences in transplant center organ acceptance practices across regions may confound the OPO behavior effects. These factors should be considered when incorporating the influence of OPO behavior on kidney nonuse in future research.

For our logistic regression models, we did not conduct a formal multicollinearity analysis such as computing variance inflation factors, as they were intentionally designed to include a small, non-redundant set of variables to mitigate collinearity concerns from the outset. We primarily assessed feature importance based on the magnitude of standardized coefficients, which allows for a meaningful comparison across variables with different scales. Additionally, clinical interpretability and relevance guided our final variable selection, particularly during the co-design phase with transplant experts. Multicollinearity analysis should be performed, particularly for automated feature selection based on logistic regression in expanded models. Our final computational results are based on the RF model that includes KDRI and nine additional variables since it achieves the best performance. Random forest models are inherently robust to multicollinearity because they rely on an ensemble of decision trees, each built on a random subset of features. This randomness reduces the dominance of correlated predictors and spreads importance across them, preventing overfitting to any single redundant variable. As a result, random forests maintain predictive accuracy even when strong correlations exist among input variables.

The application of nonuse risk prediction models must strike a balance between improving kidney utilization and safeguarding recipient outcomes. Incorporating lower-quality kidneys into the transplant pool requires careful clinical judgment to minimize the risk of post-transplant complications. Importantly, our models are not intended to make organ acceptance decisions. These decisions should remain the responsibility of transplant clinicians, made in collaboration with patients and guided by individual clinical circumstances and preferences to uphold recipient welfare.

Finally, like all predictive tools, the proposed models are subject to misclassification. Kidneys incorrectly flagged as likely to be unused may undergo unnecessary interventions, while those falsely assumed likely to be accepted may miss needed support. These risks highlight the importance of using predictive models as decision support tools to augment, not replace, clinical judgment. Operationalizing these models would require close collaboration with transplant centers, OPOs, and policymakers. One potential pathway is to integrate them into existing OPO workflows as early screening tools for identifying kidneys at high risk of nonuse that may benefit from proactive interventions. Embedding the models within platforms like DonorNet, along with clear interpretability and clinician feedback loops, could improve transparency and foster trust among users.

## 6 Conclusion

We develop interpretable, simplified models that accurately predict the nonuse risk of deceased donor kidneys and support timely, data-driven allocation interventions that can alleviate the alarming rates of kidney nonuse. Despite using a small number of variables, including KDRI and features informed by machine learning and clinical co-design, these models achieve performance comparable to that of more complex alternatives. Their integration into the organ allocation process could improve organ utilization and reduce disparities in access to transplantation. Future work should validate these models with updated datasets, assess the impact of recent policy changes, and explore their real-world implementation.

## Data Availability

Publicly available datasets were analyzed in this study. This data can be found here: United Network for Organ Sharing.
