# Supplementary material for: Improving deceased donor kidney utilization: predicting risk of nonuse with interpretable models
Source: Front Artif Intell. 2025 Aug 13;8:1638574. doi: 10.3389/frai.2025.1638574 (PMC12380666; doi:10.3389/frai.2025.1638574)
Supplement: Supplementary file 1 [file Table_1.docx]

***Table A1.*** *The original and final categories of the variables included in the analysis.*

| **Variable Name** | **Original Category** | **Count** | **Category** |
| --- | --- | --- | --- |
| Ethnicity | White | 80031 | Other |
|  | Multiracial | 1032 |  |
|  | Amer Ind/  Alaska Native | 739 |  |
|  | Native Hawaiian/  other Pacific Islander | 365 |  |
|  | Asian | 17218 | Asian |
|  | African American | 16970 | African American |
|  | Hispanic | 2979 | Hispanic |
|  |  |  |  |
| Blood Type | O | 56959 | O |
|  | A1 | 21076 | A |
|  | A | 18797 |  |
|  | A2 | 4488 |  |
|  | B | 13925 | B |
|  | AB | 1937 | AB |
|  | A1B | 1434 |  |
|  | A2B | 718 |  |
|  |  |  |  |
| Cause  of Death | Anoxia | 52334 | Anoxia |
|  | Trauma | 32836 | Trauma |
|  | CVA/Stroke | 30337 | CVA/Stroke |
|  | Other | 3426 | Other |
|  | Tumor | 401 |  |
|  |  |  |  |
| Diabetes Status | No | 105387 | No |
|  | Unknown | 1183 |  |
|  | Yes  [0-5Years] | 4914 | Yes [>0Years]  Yes [>5Years]  Yes [>10Years] |
|  | Yes  [6-10Years] | 2448 |  |
|  | Yes [>10Years] | 3819 |  |
|  | Yes [Unknown] | 1583 |  |

| **Variable Name** | **Original Category** | **Count** | **Category** |
| --- | --- | --- | --- |
| History of  Hypertension | No | 78683 | No |
|  | Unknown | 1286 |  |
|  | Yes | 39365 | Yes |
|  |  |  |  |
| History of  Cancer | No | 114554 | No |
|  | Unknown | 1172 |  |
|  | Yes | 3608 | Yes |
|  |  |  |  |
| History of Myocardial Infraction | No | 111544 | No |
|  | Unknown | 1617 |  |
|  | NaN | 1279 |  |
|  | Yes | 4894 | Yes |
|  |  |  |  |
| History of  Cigarette | No | 91202 | No |
|  | Unknown | 2650 |  |
|  | Yes | 25480 | Yes |
|  | NaN | 2 | Remove |
|  |  |  |  |
| History of  Cocaine | No | 89130 | No |
|  | Unknown | 2313 |  |
|  | NaN | 1262 |  |
|  | Yes | 26629 | Yes |
|  |  |  |  |
| History of  I.V. Drug | No | 101805 | No |
|  | Unknown | 1948 |  |
|  | Yes | 15575 | Yes |
|  | NaN | 6 | Remove |
|  |  |  |  |
| History of  Other Drug | No | 59661 | No |
|  | Unknown | 1681 |  |
|  | NaN | 1262 |  |
|  | Yes | 56730 | Yes |
|  |  |  |  |
| HCV NAT Results | Negative | 112820 | Negative |
|  | Positive | 6491 | Positive |
|  | Not Done | 12 | Remove |
|  | Indeterminant | 9 | Remove |
|  | Unknown | 2 | Remove |

| **Variable Name** | **Original Category** | **Count** | **Category** |
| --- | --- | --- | --- |
| Insulin | No | 62527 | No |
|  | NaN | 14365 |  |
|  | Yes | 42442 | Yes |
|  |  |  |  |
| HBV Core Antibody Status | Negative | 113430 | Negative |
|  | Not Done | 124 |  |
|  | Positive | 5776 | Positive |
|  | Indeterminant | 4 | Remove |
|  |  |  |  |
| Arginine Vasopressin With 24hrs pre-Clamp | Yes | 69561 | Yes |
|  | No | 48463 | No |
|  | NaN | 1274 |  |
|  | Unknown | 36 |  |
|  |  |  |  |
| Coronary  Angiogram | No | 93051 | No |
|  | NaN | 1275 |  |
|  | Yes | 25008 | Yes |
|  |  |  |  |
| Protein in Urine | Yes | 60886 | Yes |
|  | No | 56450 | No |
|  | NaN | 1262 |  |
|  | Unknown | 736 |  |
|  |  |  |  |
| Risk for Blood-Borne Disease Transmission | No | 89756 | No |
|  | Yes | 29572 | Yes |
|  | Unknown | 6 | Remove |
|  |  |  |  |
| CMV Status | Positive | 72966 | Positive |
|  | Indeterminant | 490 |  |
|  | Negative | 45794 | Negative |
|  | Not Done | 82 |  |
|  | Unknown | 2 | Remove |

| **Variable Name** | **Original Category** | **Count** | **Category** |
| --- | --- | --- | --- |
| Kidney Biopsy | Yes | 65606 | Yes |
|  | No | 50910 | No |
|  | NaN | 2818 |  |
|  |  |  |  |
| Kidney  Percentage of  Glomerulo-  sclerosis | Unknown | 51818 | 0-5 |
|  | 0-5 | 40602 |  |
|  | Not Reported | 2468 |  |
|  | Indeterminate | 280 |  |
|  | 20+ | 6409 | >20 |
|  | 6-10 | 10028 | >5 |
|  | 11-15 | 4800 | >10 |
|  | 16-20 | 2929 | >15 |
|  |  |  |  |
| Kidney  Interstitial Fibrosis | Unknown | 51805 | Absent |
|  | Absent | 27534 |  |
|  | Not Reported | 2468 |  |
|  | Unknown | 482 |  |
|  | Minimal | 17447 | Minimal |
|  | Mild | 15129 | Advanced |
|  | Mild-moderate | 3921 |  |
|  | Severe | 548 |  |
|  |  |  |  |
| Kidney  Vascular Changes | Unknown | 51807 | Absent |
|  | Absent | 32192 |  |
|  | Not Reported | 2468 |  |
|  | Unknown | 2312 |  |
|  | Minimal | 12489 | Minimal |
|  | Mild | 12263 | Advanced |
|  | Mild-moderate | 4793 |  |
|  | Severe | 1010 |  |

***Table A2.*** *Linear splines considered in the logistic regression models.*

| **Variable Name** | **Linear Spline (LS)** | |
| --- | --- | --- |
| Age | > 55 | right LS |
|  | > 65 | right LS |
| Height | < 160 | left LS |
| BMI | > 30 | right LS |
| Creatinine | > 1 | right LS |
|  | > 2 | right LS |
| WIT | > 0.5 | right LS |
|  | > 1 | right LS |
| Initial CIT | > 5 | right LS |
|  | > 10 | right LS |

*Notes. Example spline calculation: Consider age and height, which are continuous variables. Assume that we want to add the right linear spline variable at age 55. For the donor* $i$ *with age* $A_{i}$*, the right linear spline variable takes the positive part of* $\left( A_{i}-55 \right)$*. Similarly, if we want to add the left linear spine variable at height 160, the variable takes the positive part of* $\left( 160-H_{i} \right)$ *for* $H_{i}$ *denotes the donor height.*

***Table A3.*** *The number of false positives avoided by proposed prediction models without biopsy-related variables compared to using only the KDRI. Recall represents the faction of all unused kidneys that are correctly classified.*

| Recall | RF  (KDRI + 9 Variables) | LR  (KDRI + 9 Variables) |
| --- | --- | --- |
| 0.5 | 5739 | 1395 |
| 0.6 | 9078 | 2202 |
| 0.7 | 13694 | 3151 |
| 0.8 | 17136 | 5033 |

***Table A4.*** *Adjusted odds ratio for cluster indicators. The odds ratio is adjusted for the predicted risk.*

| Variable Name | Adjusted Odds Ratio for Nonuse | 95% CI |
| --- | --- | --- |
| OPO Cluster2 | 0.86 | 0.81, 0.90 |
| OPO Cluster3 | 0.81 | 0.74, 0.89 |
| OPO Cluster4 | 1.13 | 1.07, 1.18 |
| OPO Cluster5 | 1.05 | 0.85, 1.28 |


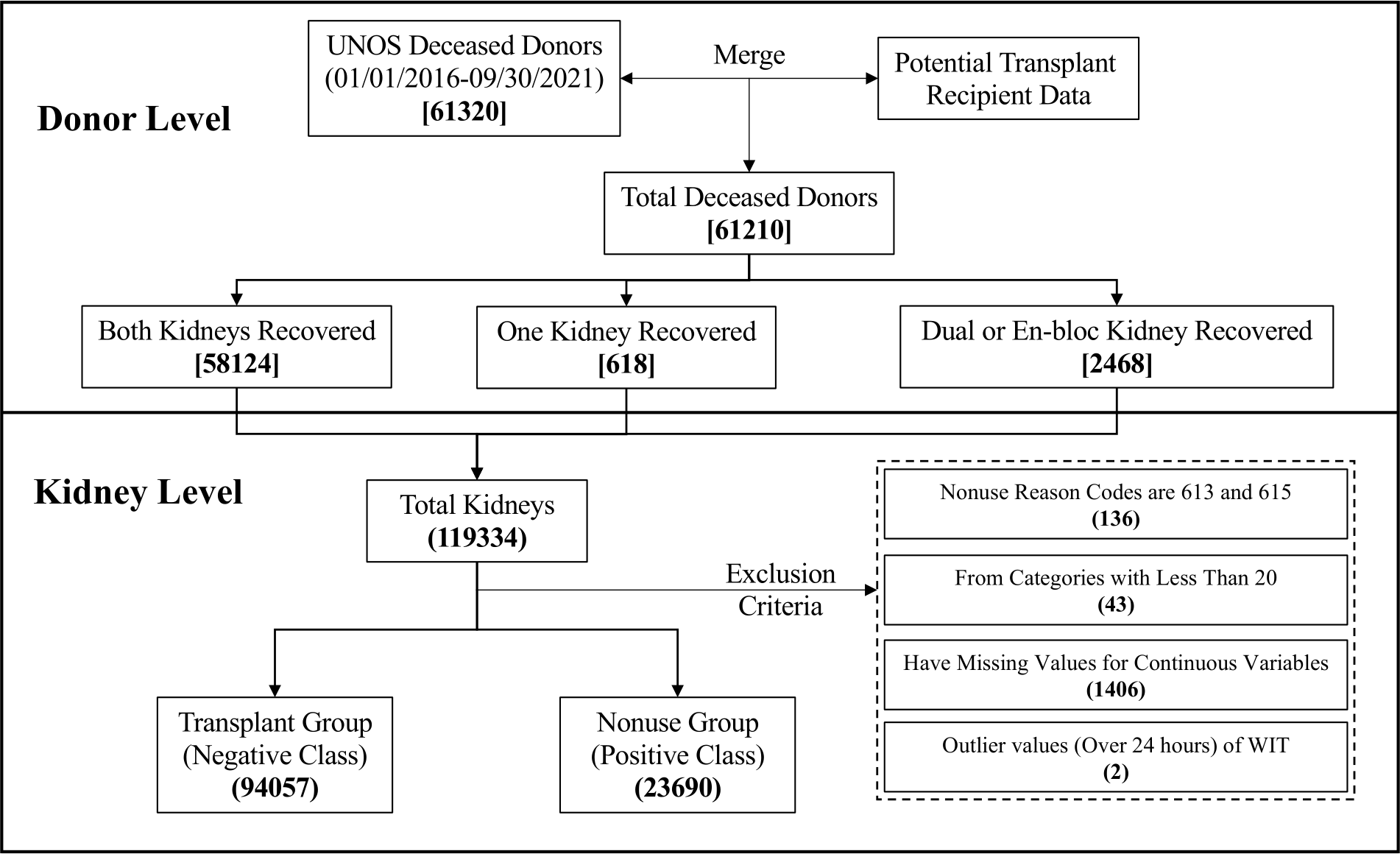


***Figure A1.*** *The process for creating the kidney-level data set. Numbers inside the square brackets and parentheses indicate the number of donors and kidneys, respectively.*

*
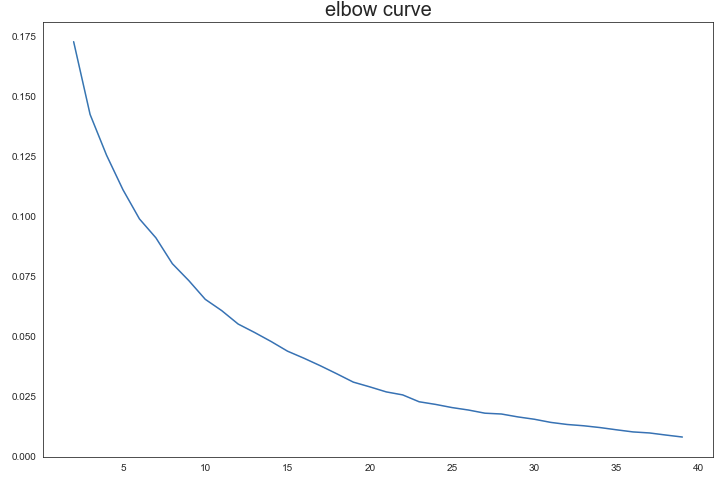
*

***Figure A2.*** *Elbow curve for K-means clustering. The x-axis represents the number of clusters (ranging from 2 to 40), and the y-axis shows the average within-cluster Euclidean distance.*

*
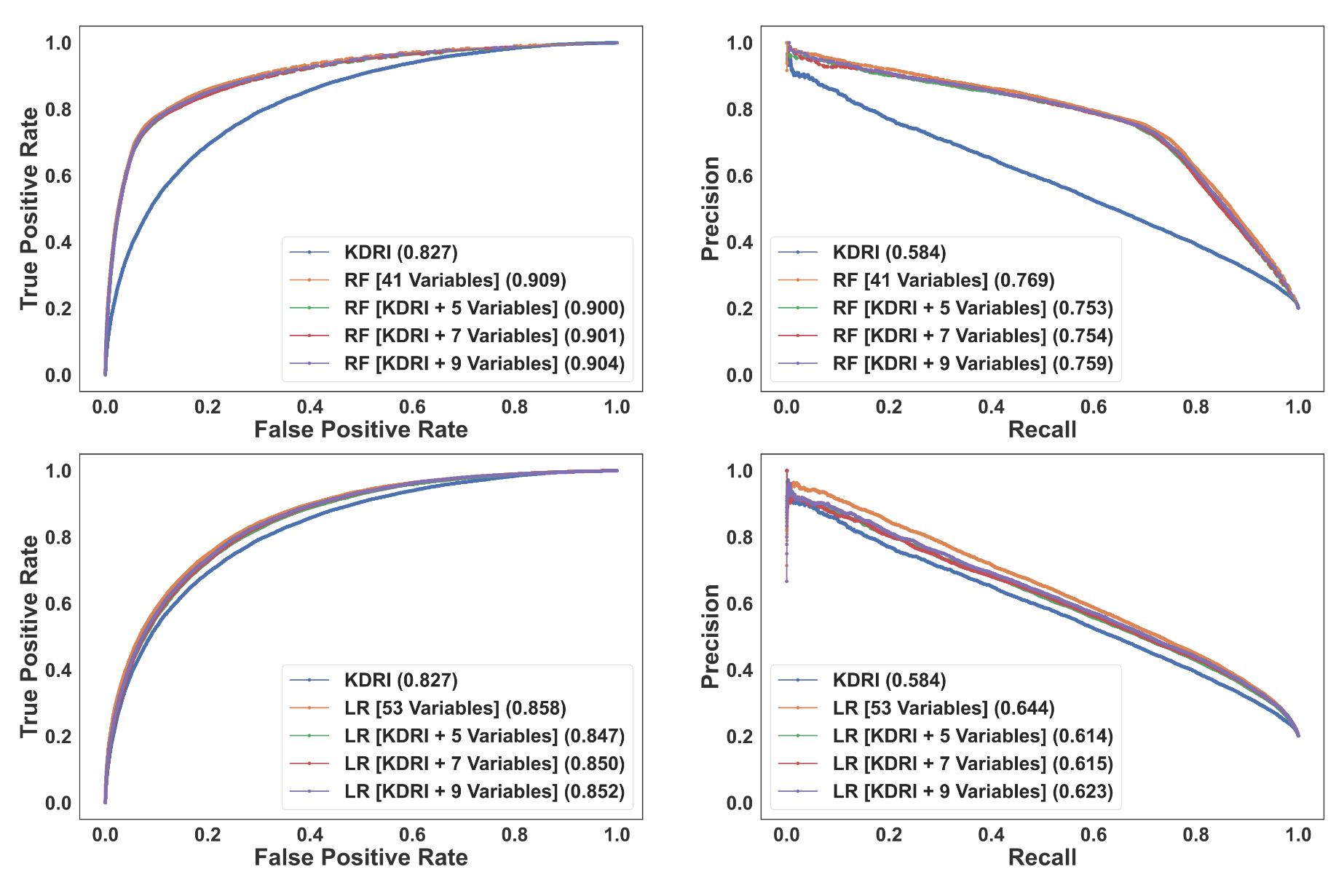
*

**Figure A3.** The ROC (left) and PR curves (right) for random forests and logistic regression models when biopsy information is unavailable. The area under the curve of each model is reported in the legend.


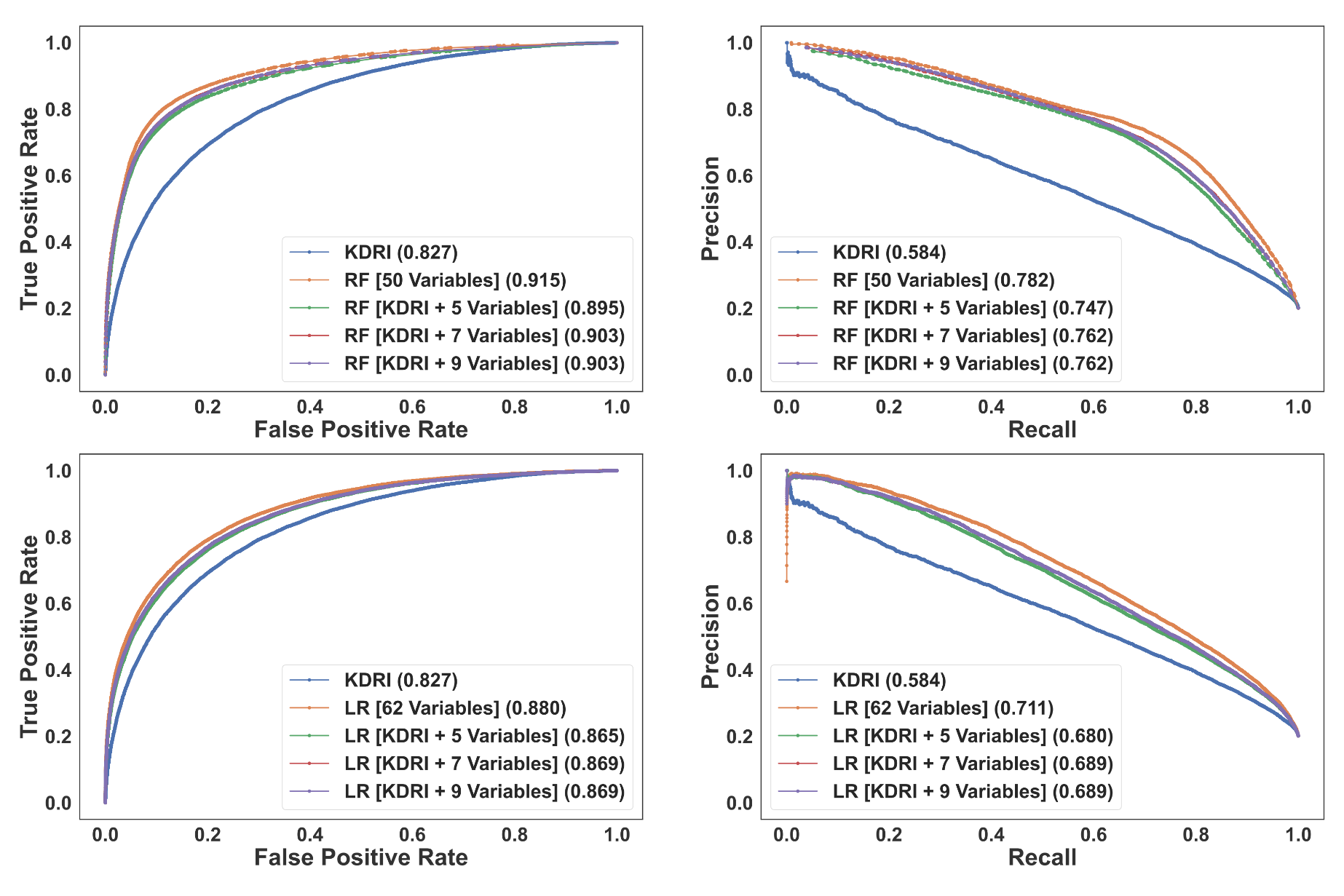


**Figure A4.** The ROC (left) and PR curves (right) for random forests and logistic regression models when biopsy information is available. The area under the curve of each model is reported in the legend.


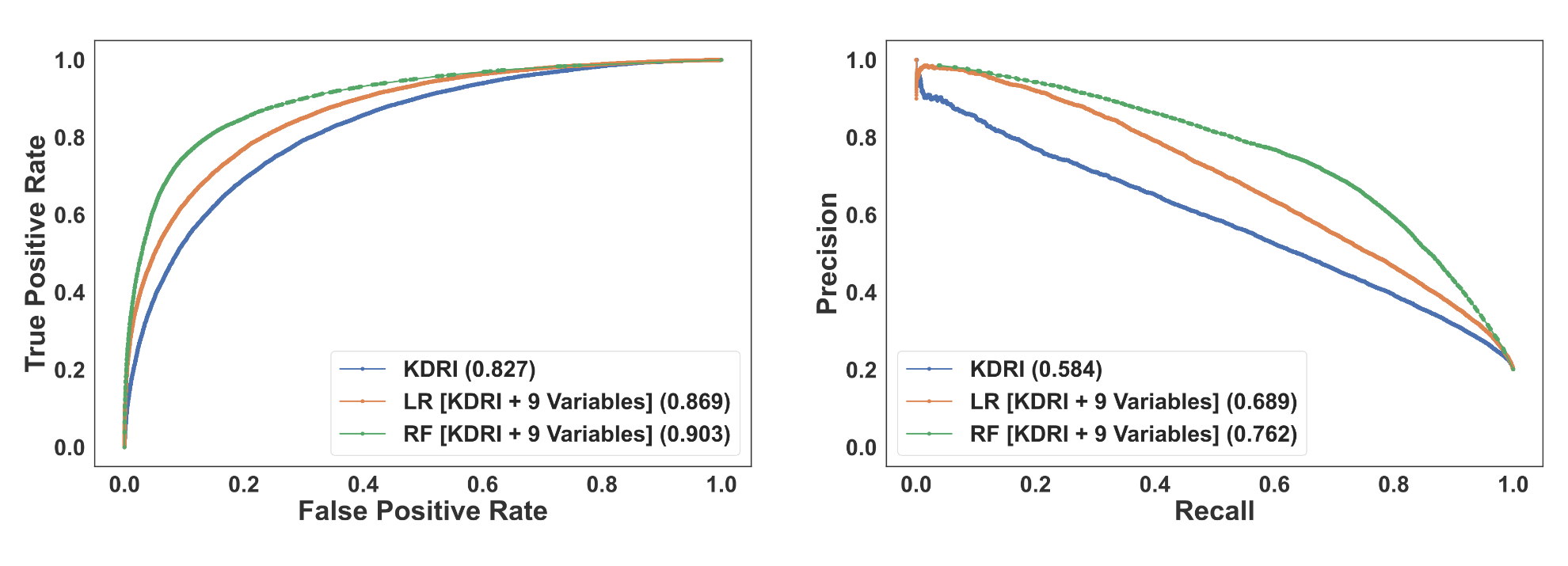


**Figure A5.** The ROC (left) and PR (right) curves for the simplified models incorporating KDRI and nine additional variables when biopsy information is available. The area under the curve of each model is reported in the legend.
